# Compound Jamming Recognition Under Low JNR Setting Based on a Dual-Branch Residual Fusion Network

**DOI:** 10.3390/s25185881

**Published:** 2025-09-19

**Authors:** Wen Lu, Junbao Li, Feng Xie, Huanyu Liu

**Affiliations:** School of Computer Science and Technology, Harbin Institute of Technology, Harbin 150001, China; 23b936024@stu.hit.edu.cn (W.L.); lijunbao@hit.edu.cn (J.L.); 21b905013@stu.hit.edu.cn (F.X.)

**Keywords:** jamming recognition, deep learning, feature fusion, dual-branch architecture

## Abstract

In complex electromagnetic environments, radar systems face increasing challenges from advanced jamming techniques. These challenges mainly stem from the diversity of jamming patterns, the complexity of compound jamming signals, and the difficulty of recognition under low jamming-to-noise ratio conditions. Accurate recognition of such signals is critical for enhancing radar anti-jamming capabilities. However, traditional methods often struggle with diverse and evolving jamming patterns. To address this issue, we propose a novel deep learning-based approach for accurate and robust recognition of complex radar jamming signals. Specifically, the proposed network adopts a dual-branch architecture that concurrently processes time-domain and time–frequency-domain features of jamming signals. It further incorporates a multi-branch convolutional structure to strengthen feature extraction and applies an effective feature fusion strategy to capture subtle patterns. Simulation results demonstrate that the proposed method outperforms six representative baseline approaches in recognition accuracy and noise robustness, particularly under low jamming-to-noise ratio conditions.

## 1. Introduction

Radar systems are primarily designed for target detection, identification, tracking, and imaging. In complex electromagnetic confrontation environments, accurately acquiring genuine target information is crucial for situational awareness and effective decision-making [1]. However, the presence of jamming signals can adversely affect these functions, posing significant challenges to radar performance. Based on their mechanisms of interference, active radar jamming signals can be broadly classified into two types: suppression jamming and deceptive jamming. Suppression jamming aims to increase the noise level at the receiver, effectively masking target echoes. In contrast, deceptive jamming attempts to mislead radar target recognition and localization by generating false echoes.

With the rapid development of radar jamming technologies, interference methods have evolved from simple single-mode jamming to more complex multi-level compound jamming. Especially in scenarios where deceptive and suppressive jamming coexist, traditional anti-jamming methods, typically designed for specific interference types, become significantly less effective in complex environments [2,3,4]. Accurate recognition of compound jamming signals is crucial for enhancing radar anti-jamming performance. It not only supports the optimization of anti-jamming strategies but also improves jamming suppression and target detection accuracy. Generally, compound jamming consists primarily of main-lobe deceptive jamming and side-lobe suppressive jamming. In typical confrontation scenarios, long-range jamming emitters weaken target detection by generating suppressive signals through the side lobes, while short-range emitters simultaneously release deceptive jamming in the main-lobe direction, misleading target recognition [5]. The combination of deceptive and suppressive interference forms a compound jamming pattern that significantly increases the difficulty of radar anti-jamming. Current radar systems typically employ adaptive beamforming as a countermeasure to mitigate side-lobe jamming; however, this approach remains ineffective against main-lobe interference [6]. Given the high energy of side-lobe suppression jamming and the pronounced deceptive characteristics of main-lobe jamming in practical scenarios, this study focuses on the identification of compound jamming composed of residual side-lobe suppression and main-lobe deception after adaptive beamforming. Consequently, a targeted recognition framework for compound radar jamming is investigated in this work.

Traditional jamming recognition methods predominantly depend on prior knowledge and expert experience to classify jamming types [7,8]. For instance, Bandiera et al. [9] proposed an adaptive detection method based on the Generalized Likelihood Ratio Test (GLRT) to classify various signal types in the presence of clutter and noise interference. However, in practical applications, acquiring accurate prior knowledge is often costly, and the dependence on expert-driven processes increases operational complexity, thereby limiting the practical applicability of these approaches. To reduce reliance on prior knowledge and expert heuristics, recent studies have proposed hand-crafted feature extraction methods combined with traditional classifiers for jamming type recognition. Techniques such as Support Vector Machines (SVMs), decision trees, and threshold-matching algorithms have been widely employed in jamming signal classification tasks [10,11,12]. For example, Liu et al. [13] extracted polarization scattering features from jamming signals and applied SVM for classification. Similarly, Su et al. [14] conducted a comparative analysis of time-domain and frequency-domain features extracted using fixed thresholds and utilized the resulting feature parameters for jamming classification. However, these methods still heavily rely on manual feature engineering, which is labor-intensive and time-consuming, thereby significantly limiting their practical deployment [15].

In recent years, deep learning-based radar jamming recognition methods have rapidly emerged, primarily driven by the powerful automatic feature extraction capabilities of Convolutional Neural Networks (CNNs) [16,17,18,19]. For instance, [20] proposed a jamming recognition network that leverages robust power spectral features by incorporating residual blocks and asymmetric convolution modules, enabling accurate identification of various suppression jamming signals. In [21], a multi-feature fusion network based on the fractional Fourier transform was developed, combining the fractional domain characteristics of jamming signals with an attention mechanism, achieving a recognition accuracy exceeding 99% at a jamming-to-noise ratio (JNR) of −3 dB. Moreover, several studies have investigated hybrid approaches that integrate sequence-based and image-based recognition methods to fully exploit the multidimensional features of jamming signals. These features are subsequently deeply fused to enhance the robustness of the recognition process. For example, [22] proposed a deep fusion CNN model that employs a 1D-CNN to extract time-domain features and a 2D-CNN to capture time–frequency-domain features derived from the short-time Fourier transform (STFT). The fusion of these feature types significantly enhanced recognition performance. Similarly, [23] developed a parallel network architecture that simultaneously processes frequency-domain feature sequences and STFT-based spectrograms as input, with 1D-CNN and 2D-CNN operating in parallel. Simulation results demonstrated that the model effectively identified 10 types of compound jamming when the JNR exceeded 0 dB. To address the integration of frequency-domain and time–frequency-domain features, [24] proposed a parallel architecture combining ResNet [25] and LSTM [26], achieving a recognition accuracy of 94.8% for six typical active jamming types. Other works have focused on recognizing jamming signals under small-sample or open-set conditions. For instance, [27] proposed a weighted ensemble CNN model based on transfer learning. It extracts time–frequency features via STFT and employs weighted voting and transfer learning techniques to improve performance with limited training samples. Additionally, [28] introduced an open-set recognition approach for jamming signals by leveraging generative adversarial networks (GANs) to generate synthetic unknown jamming samples during training, enabling the model to accurately classify known jamming while effectively rejecting unknown interference.

In the face of increasingly complex electromagnetic confrontation environments, traditional radar signal processing methods are proving inadequate to address the practical challenges posed by diverse and evolving jamming conditions. In particular, compound jamming signals often exhibit significant structural overlap and spectral similarity between their constituent components, resulting in increased feature ambiguity and reduced inter-class separability. Moreover, under low JNR conditions, the discriminative features of jamming signals are often severely obscured by noise, significantly complicating accurate recognition. To effectively counter these challenges, it is essential to fully leverage the multi-dimensional features of jamming signals and develop more efficient and robust network architectures capable of accurately recognizing and classifying both typical and compound jamming types. To this end, this paper proposes a Dual-Branch Residual Fusion Network (DBRF-Net), which concurrently exploits time-domain and time–frequency-domain features of jamming signals to enhance recognition performance under complex interference scenarios. The main contributions and innovations of this work are summarized as follows:

1. To fully exploit the information embedded in different domains of jamming signals, we propose DBRF-Net to enable the simultaneous processing of both time-domain signals and time–frequency images. Extensive recognition experiments are conducted on nine typical single jamming types and twenty compound jamming types. Compared with six representative baseline methods, the proposed method consistently achieves higher recognition accuracy across a wide range of JNRs and demonstrates robust performance even under extremely low JNR conditions.
2. To enhance the network’s ability to extract and learn task-relevant features, the proposed method replaces conventional single convolutional blocks with a diverse branch block (DBB). This module applies multi-scale convolution kernels in parallel across different branches to perform multi-scale feature extraction on the time–frequency images of jamming signals. This design significantly improves the network’s capacity to capture rich and discriminative features, thereby providing more robust representations for downstream recognition tasks.
3. To fully exploit both time-domain and time–frequency-domain information, an adaptive feature fusion module (AFFM) is designed to integrate the features extracted from the dual branches. This module performs deep fusion of time-domain and time–frequency-domain features, enabling the network to capture complementary information across modalities. Ablation experiments demonstrate that incorporating this module significantly enhances the model’s recognition performance.

The rest of this paper is organized as follows. Section 2 describes the mathematical models of both single and compound jamming types, and elaborates on the time–frequency principles of the continuous wavelet transform. Section 3 provides a detailed description of the proposed network architecture, including the DBB and AFFM. Section 4 presents a comprehensive performance comparison between the proposed method and six representative baseline methods, focusing on recognition accuracy and other key evaluation metrics. Finally, Section 5 concludes the article and outlines potential future research directions.

## 2. Modeling and Time–Frequency Analysis of Jamming Signals

In this study, several representative suppression and deceptive jamming signals are selected, and compound jamming signals are synthesized by pairing them in all possible combinations. A total of nine jamming types are considered. These include five suppression jamming methods: radio frequency noise jamming, amplitude modulation noise jamming, frequency modulation noise jamming, noise convolution jamming, and noise product jamming. In addition, four deceptive jamming methods are included: dense false target jamming, interrupted sampling direct forwarding jamming, interrupted sampling repetitive forwarding jamming, and interrupted sampling loop forwarding jamming.

The radar transmitted signal is assumed to be a linear frequency modulated (LFM) waveform, which can be mathematically expressed as:

(1) $$S\left(t\right)=A\exp\left(j2\pi f_{0}t+j\pi kt^{2}\right),$$

where $f_{0}$ denotes the carrier frequency, k represents the modulation slope, and A is the amplitude of the transmitted signal.

Based on the definitions of radar jamming signals, the following sections provide specific modeling and analysis of these signals.

### 2.1. Single Jamming Signals

1. Radio Frequency Noise (RFN) Jamming. The mathematical expression of RFN jamming is given by:

(2) $$J_{\mathrm{RFN}}\left(t\right)=U_{n}\left(t\right)\cos\left(\omega_{j}t+\phi \right),$$

where $U_{n}\left(t\right)$ represents Gaussian white noise, ϕ is a random variable following a uniform distribution $\left[0,2\pi \right)$ and is independent of $U_{n}\left(t\right)$. The carrier frequency $\omega_{j}$ is constant. Since early implementations of $J_{\mathrm{RFN}}\left(t\right)$ were primarily generated by filtering and amplifying low-power wideband analog radio frequency noise, this type of interference is also referred to as direct noise jamming.

2. Amplitude Modulation Noise (AMN) Jamming. The mathematical expression of AMN jamming is given by:

(3) $$J_{\mathrm{AMN}}\left(t\right)=(U_{0}+U_{n}\left(t\right))\cos\left(\omega_{j}t+\phi \right),$$

where $U_{0}$ and $\omega_{j}$ are constants, while $U_{n}\left(t\right),$ and ϕ retain the same definitions as previously described.

3. Frequency Modulation Noise (FMN) Jamming. The mathematical expression of FMN jamming is given by:

(4) $$J_{\mathrm{FMN}}\left(t\right)=U_{j}\cos(\omega_{j}t+2\pi K_{\mathrm{FM}}\int_{0}^{t}u\left(t^{\prime }\right)dt^{\prime }+\phi ),$$

where $u\left(t\right)$ represents Gaussian white noise, ϕ is a random variable following a uniform distribution $\left[0,2\pi \right)$ and is independent of $u\left(t\right)$, $U_{j}$, $\omega_{j}$ and $K_{\mathrm{FM}}$ are constants, representing the amplitude, carrier frequency, and frequency modulation slope of FMN, respectively.

4. Noise Convolution Jamming (NCJ). NCJ is a jamming technique where delayed radar signals are convolved with Gaussian noise, resulting in the suppression of real target signals in both the time domain and frequency domain. The mathematical expression of NCJ is given by:

(5) $$J_{\mathrm{NCJ}}\left(t\right)=S\left(t-\tau \right)⊗n\left(t\right),$$

where $S\left(t\right)$ represents the LFM signal received by the jammer, $n\left(t\right)$ denotes Gaussian white noise, “⊗” indicates the convolution operation, and τ is the time delay.

5. Noise Productive Jamming (NPJ). NPJ is a jamming technique where delayed radar signals are multiplied by Gaussian noise. The mathematical expression of NPJ is given by:

(6) $$J_{\mathrm{NPJ}}\left(t\right)=S\left(t-\tau \right)\times n\left(t\right),$$

where “×” indicates the multiplication operation.

6. Dense False Target Jamming (DFTJ). DFTJ is a jamming technique where the jammer repeatedly retransmits the radar’s transmitted signal multiple times to create high-density false targets, thereby achieving a deceptive effect. The mathematical expression of DFTJ is given by:

(7) $$J_{\mathrm{DFTJ}}\left(t\right)=\sum_{n=1}^{N}A_{i}S\left(t-\tau_{i}\right),$$

where $A_{i}$ represents the amplitude coefficient of the jamming sub-pulse, $\tau_{i}$ is the time delay interval between jamming pulses, and N denotes the number of false targets.

7. Interrupted Sampling Direct forwarding Jamming (ISDJ). ISDJ is a typical deception jamming technique based on Digital Radio Frequency Memory (DRFM) technology. The fundamental principle involves the jammer receiving the radar-transmitted echo signal and performing high-fidelity sampling on a portion of the time domain signal using a time window. During non-sampling intervals, the jammer retransmits the intercepted signal while maintaining a “sample-forward” cycle until the radar pulse ends. The mathematical expression of ISDJ is given by:

(8) $$J_{\mathrm{ISDJ}}\left(t\right)=\sum_{m=1}^{M}\mathrm{rect}\left(\frac{t-\left(2m-1\right)\tau_{j}}{\tau_{j}}\right)S\left(t-\tau_{j}\right),$$

where M represents the number of cycles, and $\tau_{j}$ denotes the sampling duration.

8. Interrupted Sampling Repetitive forwarding jamming (ISRJ). ISRJ differs from ISDJ in that, during the sampling intervals, the segmented signal is retransmitted multiple times before transitioning to the next cycle. This repetitive forwarding process continues until the entire signal reception is complete. The mathematical expression of ISRJ is given by:

(9) $$\left\{\begin{array}{l} J_{\mathrm{ISRJ}}\left(t\right)=\sum_{n=1}^{N}\sum_{m=1}^{M}S\left(t-n\tau_{j}\right)\mathrm{rect}\left(p_{\mathrm{mn}}\right)\\ p_{\mathrm{mn}}=\frac{t-\left(m-1\right)\left(N+1\right)\tau_{j}-n\tau_{j}}{\tau_{j}} \end{array}\right.,$$

where N represents the number of retransmissions for each signal segment, and M denotes the number of sampled segments.

9. Interrupted Sampling Loop forwarding Jamming (ISLJ). ISLJ is a jamming technique that follows a specific retransmission rule. After completing the n-th sampling, the sampled segments are not immediately forwarded. Instead, these segments are temporarily stored within the jammer and subsequently retransmitted over multiple time intervals according to an “integer multiple of the sampling interval” pattern. This looping process continues until the entire signal reception is complete. The mathematical expression of ISLJ is given by:

(10) $$\left\{\begin{array}{l} J_{\mathrm{ISCJ}}\left(t\right)=\sum_{m=1}^{M}\sum_{n=1}^{M-m+1}\mathrm{rect}\left(\frac{t-p_{m}\tau_{j}-q_{\mathrm{mn}}\tau_{j}}{\tau_{j}}\right)S\left(t-q_{\mathrm{mn}}\tau_{j}\right)\\ p_{m}=\frac{m\left(m+1\right)-2}{2}\\ q_{\mathrm{mn}}=\frac{\left(n-1\right)\left(2m+n+2\right)+2}{2} \end{array}\right.,$$

where M represents the number of echo sampling segments.

### 2.2. Compound Jamming Signals

As discussed in the introduction, real-world radar systems are frequently exposed to complex scenarios where residual side-lobe suppression jamming coexists with main-lobe deceptive jamming. Consequently, this study focuses on the recognition problem under the combined influence of suppression and deception jamming. To generate more challenging compound jamming signals and comprehensively evaluate the model’s recognition performance in complex environments, we select five representative suppression jamming types and four representative deception jamming types as described earlier. These types are combined using a “one suppression + one deception” strategy to construct compound jamming signals through additive superposition in the time domain. Specifically, the compound jamming signal $J\left(t\right)$ can be mathematically expressed as:

(11) $$J\left(t\right)=j_{\sup}(t)+j_{\mathrm{dec}}(t),$$

where $j_{\sup}(t)$ denotes the suppression jamming component and $j_{\mathrm{dec}}(t)$ denotes the deception jamming component. In total, 20 compound jamming types are generated by considering all possible pairwise combinations of the selected suppression and deception jamming types.

### 2.3. Time–Frequency Processing Methods

Feature extraction from jamming signals aims to uncover more salient and discriminative characteristics across multiple dimensions, thereby improving the effectiveness of subsequent analysis and recognition tasks. Among the various time–frequency analysis methods, the STFT is widely used due to its computational simplicity and clear structure, making it a common choice for time–frequency feature extraction in radar signals. However, STFT exhibits inherent limitations when analyzing complex non-stationary signals. Specifically, once the window function is fixed, the resulting time–frequency resolution also becomes fixed. This characteristic makes it challenging to capture local details simultaneously in both the time and frequency domains. Due to the inherent conflict between time and frequency resolution, STFT lacks the ability to adaptively adjust to the local characteristics of complex jamming signals, thus limiting its analytical effectiveness. To overcome these limitations, this paper introduces the Continuous Wavelet Transform (CWT) for multi-scale time–frequency feature extraction from both single and compound jamming signals, and for generating corresponding wavelet-based time–frequency images. Unlike STFT, the CWT analyzes the spectral components of a signal across multiple time scales by convolving it with a parent wavelet function that can be both translated and scaled. This approach achieves a balanced time–frequency resolution, enabling multi-scale analysis of local signal features with strong adaptability. The mathematical formulation of the CWT is given by:

(12) $$\left|W_{s}\left(a,b\right)\right|=\left|\frac{1}{\sqrt{a}}\int_{-\infty }^{+\infty }s\left(t\right)\psi^{*}\left(\frac{t-b}{a}\right)dt\right|,$$

where $s\left(t\right)$ denotes the signal to be analyzed. a and b represent the scale factor controlling frequency resolution and the translation factor controlling temporal localization, respectively. $\psi \left(t\right)$ denotes the parent wavelet function, and $\psi^{*}(t)$ represents its complex conjugate. In practice, since the CWT coefficients $W_{s}\left(a,b\right)$ are complex-valued, we take their magnitudes $\left|W_{s}\left(a,b\right)\right|$ to construct the time–frequency images. As a result, the generated time–frequency images are real-valued images. To make them suitable for classification, the images are further normalized by their maximum amplitude, so that their values are expressed as normalized amplitude (a.u.), highlighting relative patterns while reducing scale effects.

In this study, CWT is applied to 29 jamming types, including 9 single jamming signals and 20 compound jamming signals, to obtain the corresponding time–frequency images. To generate these images, we adopt the complex Morlet wavelet (cmor3-3) as the mother wavelet due to its favorable time–frequency localization properties. The scale parameter is sampled across 64 levels, providing a multi-resolution decomposition of the signals.

**Figure 1:**
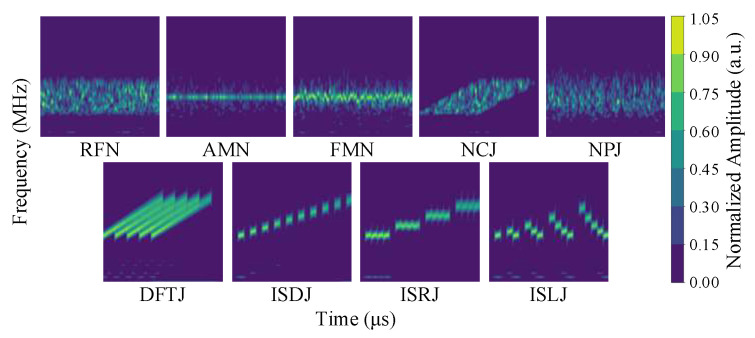
Time–frequency images of nine single jamming types obtained via CWT. The horizontal and vertical axes denote time (μs) and frequency (MHz), respectively.

Figure 1 shows the time–frequency images of the 9 single jamming signals under noise-free conditions. These signals exhibit significant differences, reflecting the distinct time–frequency characteristics of each jamming signal.

**Figure 2:**
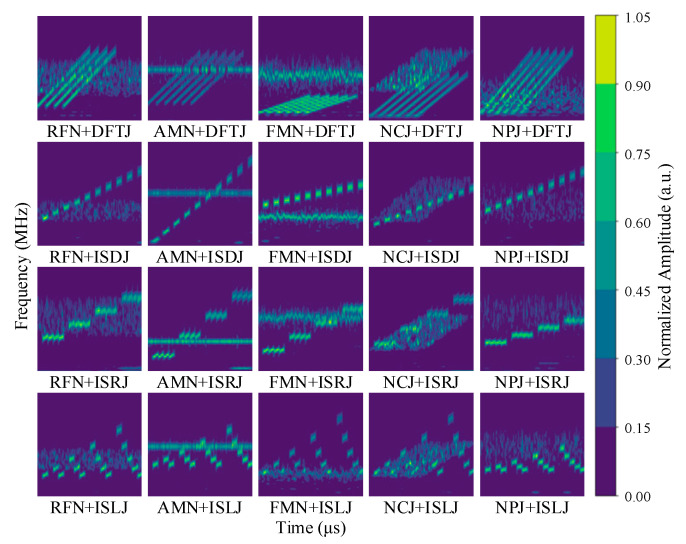
Time–frequency images of twenty compound jamming types obtained via CWT. The horizontal and vertical axes denote time (μs) and frequency (MHz), respectively.

Figure 2 shows the time–frequency images of the 20 compound jamming types under noise-free conditions. Since each compound jamming signal is generated by the additive combination of two single jamming types, it can be observed from Figure 2 that different compound jamming signals exhibit both complex structural features and distinct differences, while also sharing some similarities. Moreover, the time–frequency images show that the two single jamming components may exhibit significant overlap, which undoubtedly increases the difficulty of recognizing compound jamming. Consequently, the recognition of compound jamming is considerably more challenging compared to single jamming, placing higher demands on the model’s feature extraction capabilities, robustness, and ability to process high-dimensional data.

### 2.4. Translation Support Statement

The manuscript was originally written by the author in Chinese. During the translation process, Youdao Translator (NetEase, Hangzhou, China) was only used as an auxiliary tool for certain parts. After the translation was completed, the translated sections as well as the entire manuscript were carefully reviewed, revised, and finalized manually by the author.

## 3. Dual-Branch Residual Fusion Network (DBRF-Net)

This section first introduces the overall architecture of the proposed network, which adopts a dual-branch structure to process the time-domain and time–frequency-domain information of jamming signals in parallel. Next, the DBB [29] is described in detail. This module integrates multi-scale convolution kernels to enhance the model’s ability to extract and represent time–frequency features. Finally, the AFFM is presented, performing adaptive weighted fusion of the features extracted by the two branches, thereby effectively improving the recognition performance of the model.

### 3.1. Network Architecture

**Figure 3:**
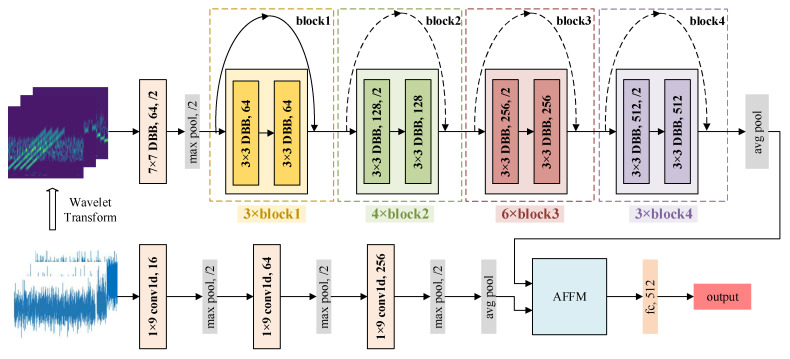
Overall architecture of the proposed DBRF-Net, which consists of a time-domain branch, a time–frequency branch, and an Adaptive Feature Fusion Module (AFFM).

To fully exploit the multi-domain information of jamming signals, this paper proposes DBRF-Net, which consists of two parallel branches. These branches are designed to process the one-dimensional time-domain signal sequence and its corresponding time–frequency image, respectively, thereby enabling joint modeling of time-domain and time–frequency-domain features. The time-domain branch directly performs convolution operations on the raw one-dimensional time-domain sequence to extract temporal variation features. In contrast, the time–frequency branch takes the time–frequency image as input and employs a residual structure to alleviate the vanishing gradient problem during deep network training, preserving more critical features. Additionally, this branch further integrates the DBB module to facilitate deep extraction and aggregation of multi-scale time–frequency features, thereby enhancing the network’s capability to represent complex jamming signals. After the dual-branch feature extraction, AFFM is introduced at the end of the network to perform deep fusion of time-domain and time–frequency-domain features. The fusion weights are learned adaptively, allowing the model to dynamically adjust the contribution of each feature component to the final classification decision. This design further improves the recognition accuracy in complex jamming scenarios, ultimately enabling high-precision identification of diverse jamming signals. The overall architecture of the proposed DBRF-Net is shown in Figure 3.

The dual-branch structure of the network fully leverages the complementary feature information of jamming signals from different domains. The time-domain branch directly processes the raw one-dimensional signal sequence, capturing amplitude variations, edge transitions, and other temporal characteristics. This branch exhibits stronger sensitivity to bursty and transient interference. In contrast, the time–frequency branch takes the wavelet-transformed time–frequency image as input. It extracts localized variation patterns within the time–frequency plane, effectively representing the spectral structure, dynamic frequency distribution, and abrupt changes in instantaneous frequency of jamming signals. By parallel extraction of time-domain and time–frequency-domain features, the dual-branch structure fuses complementary information from both domains. This structure effectively mitigates the limitations of single-domain representation, enabling deep modeling of complex jamming patterns in a broader feature space. Consequently, the dual-branch structure of the network significantly improves recognition accuracy and robustness when dealing with complex jamming signals.

Structurally, the time-domain branch primarily consists of stacked layers of one-dimensional convolutions, ReLU activation functions, and max-pooling layers, aiming to model the temporal variation patterns of jamming signals. This branch directly processes the complex-valued radar signal input, performing feature extraction separately on the real and imaginary components of the signal. Specifically, the complex signal is first decomposed into two independent one-dimensional sequences, corresponding to the real part and imaginary part. These sequences are then fed into two parallel sub-paths with identical structures but non-shared parameters. Each sub-path performs multi-layer 1D convolutional operations to extract its respective feature representation. Afterward, the features from both paths are concatenated along the feature dimension, resulting in a 512-dimensional time-domain feature vector.

The time–frequency branch takes the two-dimensional time–frequency images obtained from the CWT as input. It sequentially passes through multiple DBB convolution modules and residual connection structures to perform deep extraction of time–frequency features, ultimately producing a 512-dimensional time–frequency feature vector. As shown in Figure 3, the time–frequency branch contains four residual modules, named block1 to block4. While their internal structures are largely consistent, the differences lie in the number of repetitions and the types of residual connections used. Specifically, block1 is repeated 3 times, block2 is repeated 4 times, block3 is repeated 6 times, and block4 is repeated 3 times. In block2, block3, and block4, the first residual unit employs a dashed residual connection. This connection adjusts the shape of the input feature map by halving its height and width. It also modifies the channel depth to match the requirements of the subsequent residual structure, achieving dimensional alignment. The subsequent residual units within these blocks utilize standard residual connections, which do not alter the shape of the feature map.

After completing feature extraction in both the time domain and time–frequency domain, the network introduces AFFM. This module automatically adjusts the weight ratio of these features in the final classification decision, achieving weighted fusion at the feature level. The resulting 512-dimensional fused feature vector is then processed through a fully connected layer, ultimately producing the jamming classification result.

### 3.2. Diverse Branch Block (DBB)

Compared with many computer vision tasks, the inter-class features of radar jamming signals often exhibit high similarity and low discriminability. Therefore, in jamming recognition tasks, it is critical to design neural network architectures with enhanced feature representation capacity and fine-grained discrimination ability. Such designs enable the network to capture subtle differences between signal types, thereby improving classification performance.

**Figure 4:**
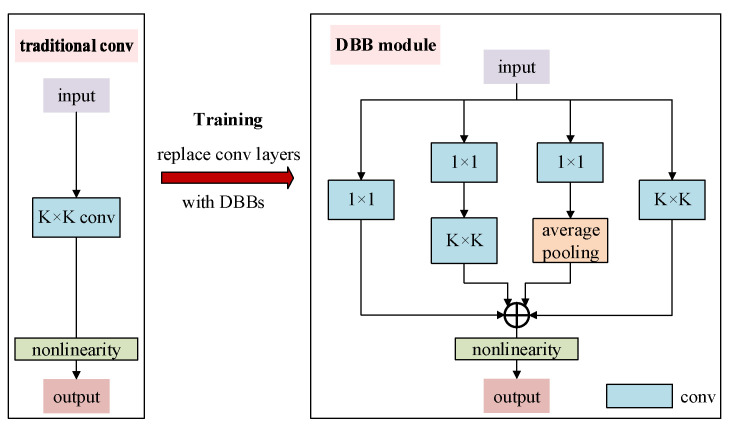
Structure of the DBB module.

To strengthen the modeling capability of the network in capturing the complex time–frequency features of jamming signals, the DBB module is incorporated into the time–frequency branch of the proposed network. During training, the DBB integrates multiple convolutional branches with different receptive fields and structural paths, significantly enhancing the representational power of convolutional layers. During inference, these branches can be equivalently merged into a standard convolution layer, ensuring that the computational cost and inference efficiency remain unchanged at deployment stage. Specifically, the DBB module enriches the feature space by combining multiple branches with varying scales and complexities, including convolutional sequences, multi-scale convolutions, and average pooling. This design enhances the expressiveness of a single convolution operation and improves the network’s ability to capture subtle differences in the time–frequency images. Consequently, it increases both the recognition accuracy and robustness in complex jamming scenarios. The structure of the DBB module is illustrated in Figure 4. For clarity of presentation, the batch normalization layers following each convolution and average pooling layer in the DBB module are omitted from the figure.

To theoretically derive the structural reparameterization of the DBB module during the inference phase, we start by defining the input feature map as $I\in {\mathbb{R}}^{C\times H\times W}$, where C denotes the number of input channels, and H and W represent the length and width of the input feature map, respectively. The parameters of a conv layer with C input channels, D output channels and kernel size K×K reside in the conv kernel, which is a 4th-order tensor $F\in {\mathbb{R}}^{D\times C\times K\times K}$, and an optional bias $b\in {\mathbb{R}}^{D}$. The corresponding output feature map is denoted as $O\in {\mathbb{R}}^{D\times H^{\prime }\times W^{\prime }}$, where D represents the number of output channels, and $H^{\prime }$ and $W^{\prime }$ are the length and width of the output feature map, respectively. Formally, the output of each convolutional branch in the network can be defined as follows:

(13) $$O=I⊗F+\mathrm{REP}\left(b\right),$$

where “⊗” denotes the convolution operation and $\mathrm{REP}\left(b\right)\in {\mathbb{R}}^{D\times H^{\prime }\times W^{\prime }}$ means replicating b to match the output dimensions. At the element level, the output $O_{j,h,w}$ on the j-th output channel at position $\left(h,w\right)$ is calculated as:

(14) $$O_{j,h,w}=\sum_{c=1}^{C}\sum_{u=1}^{K}\sum_{v=1}^{K}F_{j,c,u,v}X{\left(c,h,w\right)}_{u,v}+b_{j},$$

where $X\left(c,h,w\right)\in {\mathbb{R}}^{K\times K}$ denotes the local sliding window of the input feature map I from the c-th channel, corresponding to position (h,w) on the output feature map O. Such a correspondence is determined by the padding and stride settings. Equation (14) formulates the convolution process as a weighted summation of the inputs, indicating that convolution is essentially a linear operation. Therefore, it satisfies some fundamental properties of linear operations, including homogeneity and additivity. The homogeneity of the convolution can be defined as follows:

(15) $$I⊗\left(pF\right)=p\left(I⊗F\right),\forall p\in \mathbb{R},$$

indicating that scaling the kernel by a scalar factor is equivalent to scaling the output feature map. Furthermore, when two convolution kernels $F^{\left(1\right)}$ and $F^{\left(2\right)}$ have identical configurations (e.g., number of channels, kernel size, stride, padding, etc.), the convolution operation satisfies the additivity property, which can be defined as follows:

(16) $$I⊗F^{\left(1\right)}+I⊗F^{\left(2\right)}=I⊗\left(F^{\left(1\right)}+F^{\left(2\right)}\right).$$

Based on the homogeneity and additivity properties of convolution, the DBB module enables structural re-parameterization during the inference phase. The multi-branch convolutional structure constructed during training can be transformed into an equivalent single-branch convolution through a series of linear combinations. This transformation allows the model to be deployed in an equivalent convolutional form during inference. Such a mechanism preserves the recognition performance achieved during training while significantly reducing computational overhead and parameter redundancy at inference time. As a result, it greatly enhances the model’s deployment efficiency and inference speed.

### 3.3. Adaptive Feature Fusion Module (AFFM)

**Figure 5:**
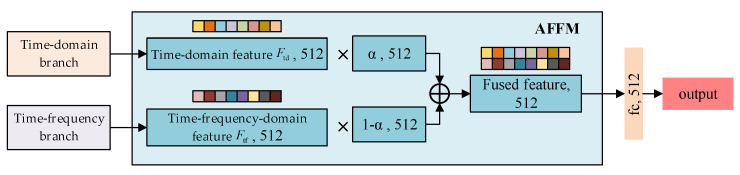
Structure of the AFFM.

To fully leverage the complementary advantages of time-domain and time–frequency-domain features in representing jamming signals, this paper designs AFFM. It efficiently fuses multi-domain features, thereby enhancing the model’s jamming recognition performance under complex environmental conditions. The structure of the AFFM is shown in Figure 5.

Unlike simple feature concatenation or average fusion, AFFM introduces a learnable weighting mechanism for feature fusion. Specifically, AFFM first flattens the time-domain feature vector $F_{\mathrm{td}}$ and the time–frequency-domain feature vector $F_{\mathrm{tf}}$, ensuring that both have consistent dimensions. The lengths of $F_{\mathrm{td}}$ and $F_{\mathrm{tf}}$ are both 512-dimensional. Next, AFFM introduces a learnable fusion weight vector α, also of 512 dimensions, to adaptively adjust the contribution of each domain’s feature in the final fused representation. This approach enables a more effective feature-level weighted fusion. The resulting fused feature vector $F_{\mathrm{fusion}}\in {\mathbb{R}}^{512}$ is computed as follows:

(17) $$F_{\mathrm{fusion}}=\alpha ⊙F_{td}+(1-\alpha )⊙F_{\mathrm{tf}},$$

where ⊙ denotes element-wise multiplication. This fusion strategy allows the model to dynamically adjust the contribution ratio of time-domain features and time–frequency features in a task-driven manner, fully leveraging the complementarity between the two types of features. Consequently, it significantly enhances the model’s ability to recognize complex jamming categories.

## 4. Experiments and Results

This section first introduces the simulated radar jamming signal dataset. Then, the experimental settings and evaluation metrics are described in detail. Subsequently, we present a quantitative comparative analysis between the proposed DBRF-Net method and existing approaches. Finally, ablation studies are conducted to further validate the effectiveness of the key modules within the proposed network.

### 4.1. Description of the Jamming Dataset

Due to the difficulty of obtaining real radar jamming signals in actual electronic countermeasure scenarios, this study simulates a representative radar jamming dataset with reference to the existing literature [20,30,31]. The simulations include the nine typical single jamming types and twenty compound jamming types introduced in Section 2. The corresponding simulation parameters are summarized in Table 1. For compound jamming signals, the simulation parameters are consistent with those of the constituent single jamming components. Moreover, in practical electronic countermeasure scenarios, jammers typically interfere with radar detection by transmitting high-power deception or suppression signals, effectively preventing the radar from detecting true target echoes. As a result, the power of target echoes is often much lower than that of the jamming signals. Furthermore, the primary objective of jamming recognition is to accurately identify the type of jamming signal so that appropriate countermeasures can be applied. Therefore, in designing the radar received signal model, this study considers only the jamming signals, which is consistent with the approaches adopted in [20,30,31].

Based on the jamming parameter settings provided in Table 1, independent datasets for the nine single jamming types and twenty compound jamming types are constructed, with the sampling frequency uniformly set to 200 MHz in all simulations. These datasets are designed to simulate various interference scenarios that radar systems may encounter in typical electronic countermeasure environments. Each jamming sample consists of 4000 complex-valued sampling points (i.e., 4000 real and 4000 imaginary values). Additionally, to better simulate the intensity variability of jamming signals and energy attenuation during spatial propagation in complex electromagnetic environments, additive white Gaussian noise is added to each original jamming sample. This allows the construction of datasets under different JNR conditions. In this study, the JNR is defined as the ratio of the jamming signal power to the noise power after matched filtering at the radar receiver, following standard practice in radar signal processing. Specifically, it is expressed as:

(18) $$\mathrm{JNR}=10\log_{10}\frac{P_{\mathrm{jam}}}{P_{\mathrm{noise}}},$$

where $P_{\mathrm{jam}}$ denotes the residual power of the jamming signal after receiver-side filtering, and $P_{\mathrm{noise}}$ denotes the corresponding noise power. While this definition approximates the interference-to-noise level at the radar receiver, the simulation framework directly controls the JNR by adjusting the power ratio between the simulated jamming signal and additive white Gaussian noise, providing a practical proxy for controlled experimentation.

The single jamming dataset consists of the time-domain sequences and their corresponding wavelet-based time–frequency images for 9 single jamming types. Considering that single jamming classification is relatively less challenging, the JNR range for this dataset is set from −20 dB to 5 dB, with a particular focus on evaluating the model’s performance under extremely low JNR conditions. To balance the diversity of sample distribution and training efficiency, training data are selected at 5 dB intervals within the −20 dB to 5 dB range, with 200 samples per jamming type at each JNR level. Similarly, the validation set uses the same JNR levels and sampling intervals, with 50 samples per type per JNR. For a comprehensive assessment of the model’s generalization performance under varying JNR conditions, the test set includes samples at 1 dB intervals across the same JNR range, with 50 samples per jamming type at each JNR level.

**Figure 6:**
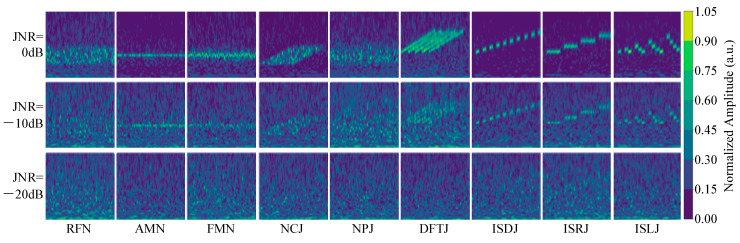
Time–frequency images of 9 single jamming types under JNRs of 0 dB, −10 dB, and −20 dB.

Figure 6 presents the time–frequency images of the 9 single jamming types under JNR conditions of 0 dB, −10 dB, and −20 dB. It can be observed that as the JNR decreases, the visual features of the jamming signals in the time–frequency images gradually weaken, while the masking effect of noise becomes increasingly prominent. At 0 dB, although the noise component is already noticeable, the structural features of the jamming signals remain partially distinguishable. When the JNR drops to −10 dB, the primary features of the jamming signals are partially obscured by noise, making some types difficult to distinguish visually. At the extremely low JNR of −20 dB, the jamming energy is severely attenuated, and the signal features are almost completely submerged in noise, resulting in an image that is clearly dominated by noise. Evidently, jamming recognition under such low-JNR conditions presents significant challenges, especially for methods that rely on visual inspection or hand-crafted feature extraction. Therefore, employing deep neural networks with strong feature abstraction capabilities becomes particularly important. Notably, the proposed DBRF-Net effectively captures weak structural features of jamming signals even in severely noisy environments, achieving high recognition accuracy while demonstrating strong robustness and anti-jamming performance.

The compound jamming dataset consists of the time-domain sequences and their corresponding wavelet-based time–frequency images for 20 compound jamming types. Since each compound jamming signal is constructed by the superposition of two different jamming types, the signal structure becomes more complex and the feature distribution more diverse, resulting in significantly greater recognition difficulty compared to single jamming. To ensure that the model can effectively learn discriminative features of compound jamming during training, while also avoiding degraded training performance caused by severe signal distortion at extremely low JNRs, the JNR range is set to −10 dB to 10 dB. This range covers typical electronic countermeasure scenarios, spanning from low to medium and high jamming intensities. It also strikes a practical balance between signal perceptibility and modeling feasibility. To balance the diversity of sample distribution with training efficiency, training data are selected at 5 dB intervals within the −10 dB to 10 dB range, with 200 samples per jamming type at each JNR level. Similarly, the validation set uses the same JNR levels and sampling intervals, with 50 samples per type per JNR. For a comprehensive assessment of the model’s generalization performance under varying JNR conditions, the test set includes samples at 1 dB intervals across the same JNR range, with 50 samples per jamming type at each JNR level.

**Figure 7:**
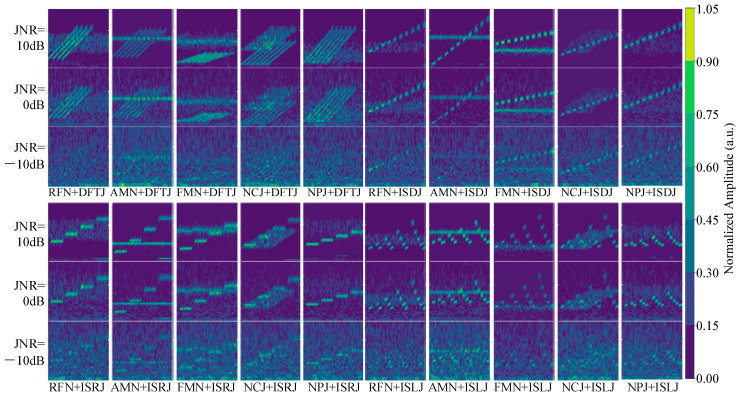
Time–frequency images of 20 compound jamming types under JNRs of 10 dB, 0 dB, and −10 dB.

Figure 7 shows the time–frequency images of the 20 compound jamming types at JNR levels of 10 dB, 0 dB, and −10 dB. It can be observed that compared to single jamming, compound jamming signals exhibit more complex and diverse structural features in the images. Their spectral distributions show stronger local non-stationarity and display evident composite features resulting from the superposition of different jamming types. This increased structural complexity significantly raises the difficulty of classification and imposes higher demands on the network’s feature learning capacity, especially under low JNR conditions. Specifically, at −10 dB, the time–frequency images are heavily dominated by noise, with jamming features severely obscured. Under such circumstances, it becomes nearly impossible to visually detect the presence of jamming, highlighting the significant challenge of compound jamming recognition in low-JNR scenarios.

### 4.2. Experimental Settings and Evaluation Metrics

To evaluate the effectiveness and robustness of the proposed DBRF-Net in jamming recognition tasks, six representative deep learning models are selected for comparative experiments. These models include 1D-CNN, 2D-CNN [32], Transformer [33], ResNet34 [25], JRNet [20], and DFCNN [22]. Among them, the 1D-CNN and Transformer models take the one-dimensional time-domain sequence of the jamming signal as input. The 2D-CNN, ResNet34, and JRNet models use the wavelet-based time–frequency images as input. Both DFCNN and the proposed DBRF-Net utilize dual inputs: the one-dimensional time-domain signal and the corresponding time–frequency image.

All experiments are conducted on a software platform consisting of Python 3.9, PyTorch 2.0, and CUDA 11.8. The hardware platform includes an AMD Ryzen 7950X CPU and an NVIDIA RTX 4090 GPU.

All models are trained under a unified configuration: the initial learning rate is set to 0.001 with a dynamic learning rate adjustment strategy during training. The batch size is 64, and the total number of training epochs is 100. Early stopping is applied if the validation loss does not significantly decrease for 7 consecutive epochs. The optimizer used is Adam [34], and the loss function is categorical cross-entropy. Additionally, the 512-dimensional learnable weight vector α in the AFFM is initialized to 0.5 and updated adaptively during training.

To comprehensively evaluate the model’s performance in the jamming recognition task, three evaluation metrics are adopted: Overall Accuracy (OA), Macro-averaged F1 score (Macro-F1), and the Kappa coefficient (Kappa). Specifically, OA measures the overall classification accuracy across the entire test set, reflecting the model’s global recognition capability. Macro-F1 calculates the unweighted average of F1 scores across all classes, highlighting the model’s ability to maintain balanced performance across different jamming types. The Kappa further evaluates the consistency between predicted and true labels by considering the level of agreement that could be expected purely by random guessing, thus providing a more robust evaluation than OA in multi-class settings. Together, these three metrics evaluate the model’s recognition and generalization performance from the perspectives of global accuracy, class-level balance, and prediction consistency, offering a well-rounded assessment under a balanced dataset setting.

Assume that $N_{c}$ denotes the total number of classes, and N represents the total number of samples. Let ${\mathrm{TP}}_{i}$, ${\mathrm{FP}}_{i}$, ${\mathrm{FN}}_{i}$ and ${\mathrm{TN}}_{i}$ denote the number of true positive (TP), false positive (FP), false negative (FN), and true negative (TN) samples for the i-th class, respectively. Then, the OA is defined as follows:

(19) $$\mathrm{OA}=\frac{\sum_{i=1}^{N_{c}}{\mathrm{TP}}_{i}}{\sum_{i=1}^{N_{c}}\left({\mathrm{TP}}_{i}+{\mathrm{FP}}_{i}+{\mathrm{FN}}_{i}\right)},$$

The Macro-F1 score is defined as follows:

(20) $$\left\{\begin{array}{l} \text{Macro-F1}=\frac{1}{N_{c}}\sum_{i=1}^{N_{c}}\frac{2\cdot {\mathrm{Precision}}_{i}\cdot {\mathrm{Recall}}_{i}}{{\mathrm{Precision}}_{i}+{\mathrm{Recall}}_{i}}\\ {\mathrm{Precision}}_{i}=\frac{{\mathrm{TP}}_{i}}{{\mathrm{TP}}_{i}+{\mathrm{FP}}_{i}}\\ {\mathrm{Recall}}_{i}=\frac{{\mathrm{TP}}_{i}}{{\mathrm{TP}}_{i}+{\mathrm{FN}}_{i}} \end{array}\right.,$$

The Kappa coefficient is defined as follows:

(21) $$\left\{\begin{array}{l} \mathrm{Kappa}=\frac{\mathrm{OA}-p_{e}}{1-p_{e}}\\ p_{e}=\sum_{i=1}^{N_{c}}\left(\frac{\left({\mathrm{TP}}_{i}+{\mathrm{FP}}_{i}\right)\cdot \left({\mathrm{TP}}_{i}+{\mathrm{FN}}_{i}\right)}{N^{2}}\right) \end{array}\right.,$$

### 4.3. Single Jamming Recognition

Based on the previously introduced single jamming dataset, we conducted recognition experiments for the single jamming scenario. The recognition accuracy of each method on the test set across different JNR levels is presented in Table 2. Overall, the proposed DBRF-Net consistently outperforms all comparison models across the full JNR range from −20 dB to 5 dB. It achieves an average accuracy of 89.7%, exceeding the second-best method, DFCNN (83.6%), by 6.1 percentage points, demonstrating superior global performance and robustness. Notably, DBRF-Net shows significant advantages in the extremely low JNR range (−20 dB to −10 dB). At −20 dB, it achieves a recognition accuracy of 53.1%, substantially outperforming Transformer (18.0%) and ResNet34 (25.8%). Moreover, its recognition accuracy increases sharply within this extremely low JNR interval, rising from 53.1% at −20 dB to 93.1% at −10 dB, highlighting its excellent noise-resilient learning capability. Further analysis reveals that DBRF-Net surpasses 90% accuracy as early as −12 dB, and approaches 99.0% at −6 dB, significantly ahead of all other comparison methods.

**Figure 8:**
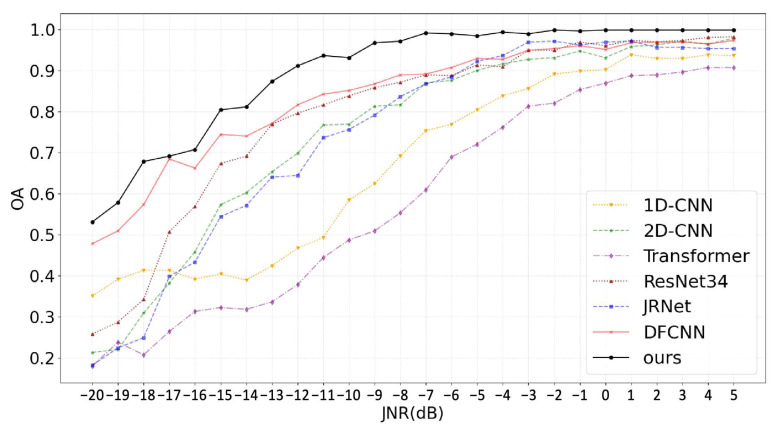
OA curves of various methods for single jamming recognition under different JNR conditions.

Figure 8 illustrates the trend curves of OA with respect to JNR for all methods on the test set from the single jamming dataset. Overall, the recognition performance of all methods improves significantly as the JNR increases, exhibiting a consistent upward trend. However, in the extremely low JNR range (−20 dB to −10 dB), performance differences among models become more pronounced, with robustness to noise playing a critical role. As the JNR gradually increases, the performance gap between models narrows. By 4 dB, all models achieve recognition accuracy above 90%, indicating that under high-JNR conditions, the features of single jamming signals become more distinguishable, enabling most models to recognize accurately. This also reflects the relatively low difficulty of single jamming recognition under such conditions. The proposed DBRF-Net consistently maintains the best performance across the entire JNR range and reaches saturation around −7 dB, suggesting that it can reliably complete the classification task under low JNR conditions. In contrast, DFCNN ranks second but still lags behind DBRF-Net by approximately 3% to 10% in accuracy. Additionally, traditional image-based models (2D-CNN, ResNet34, and JRNet) exhibit relatively poor performance in the −20 dB to −14 dB range, indicating their sensitivity to noise and limited feature extraction capabilities in low JNR environments.

It is worth noting that in the −20 dB to −18 dB range, the top three models in terms of accuracy are DBRF-Net, DFCNN, and 1D-CNN, all of which leverage time-domain signal information. Among them, 1D-CNN even outperforms several image-based models under extremely noisy conditions. This observation suggests that even weak patterns in raw time-domain signals may still retain discriminative features, while converting them into time–frequency images may amplify noise and exacerbate information loss. In contrast, DBRF-Net adopts a dual-branch architecture to extract and fuse features from both the time domain and the time–frequency domain, enabling joint exploitation of discriminative information across different representation spaces. As a result, the model can effectively extract key jamming features and maintain robust recognition performance even under severe noise conditions. These results further confirm the effectiveness and architectural advantages of the proposed dual-modality feature fusion strategy in low-JNR recognition scenarios.

Table 3 presents the comparison results of different methods on the test set from the single jamming dataset in terms of OA, Macro-F1, and Kappa. Overall, the proposed DBRF-Net achieves the best performance across all three metrics, with an OA of 89.7%, a Macro-F1 of 0.897, and a Kappa of 0.884, consistently outperforming the other methods. DFCNN ranks second in all metrics, reaching 83.6%, 0.836, and 0.816, respectively. In contrast, traditional single-branch models such as ResNet34 and JRNet achieve OA scores of 79.1% and 74.1%, respectively, indicating a considerable performance gap. Further analysis reveals that the Transformer model yields the lowest scores across all three metrics, with an OA of 58.3%, a Macro-F1 of 0.576, and a Kappa of 0.534. This may be attributed to its limited capacity to model jamming signals when the sample size is small or when strong noise obscures the distribution of discriminative features. In contrast, DBRF-Net not only maintains a high overall accuracy but also achieves a significantly higher Macro-F1, indicating balanced classification performance across all categories without neglecting any specific class. Moreover, the high Kappa score indicates that DBRF-Net’s predictions remain closely aligned with the ground truth beyond chance agreement, further validating the reliability and robustness of the proposed method.

In summary, DBRF-Net demonstrates superior performance in terms of accuracy, class-wise balance, and predictive consistency, highlighting its clear advantage in single jamming recognition tasks.

### 4.4. Compound Jamming Recognition

Based on the previously introduced compound jamming dataset, we conducted recognition experiments for the compound jamming scenario. The recognition accuracy of each method on the test set across different JNR levels is presented in Table 4. Overall, the proposed DBRF-Net outperforms all comparison models across the full JNR range of −10 dB to 10 dB, achieving an average accuracy of 90.8%, which is 7.4 percentage points higher than the second-best method, JRNet (83.4%). This result highlights the model’s strong representation capability and stable recognition performance even in complex compound jamming scenarios. Meanwhile, the proposed method achieves an accuracy of 70.5% even under a low JNR of −10 dB, surpasses 90% at −2 dB, and reaches 99% at 6 dB, maintaining a clear lead over all comparisons. Among the baselines, JRNet performs relatively well and approaches DBRF-Net under mid-to-high JNR conditions, but suffers a noticeable performance drop under low JNR levels. ResNet34 and 2D-CNN rely heavily on time–frequency images and exhibit overall stable performance. However, their single-modality input limits their ability to effectively capture the multidimensional features of compound jamming. In contrast, DBRF-Net employs a dual-branch architecture to simultaneously process time-domain and time–frequency-domain features. It also incorporates the previously introduced AFFM to effectively integrate multi-domain features. These designs enable the model to maintain strong discriminative capability even when confronted with structurally complex compound jamming signals.

**Figure 9:**
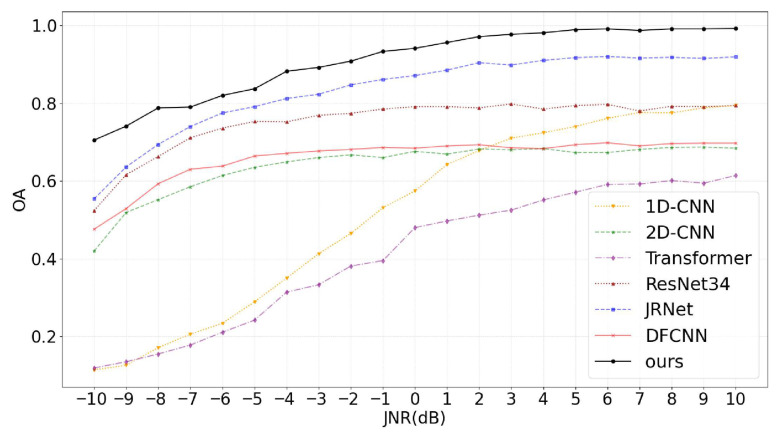
OA curves of various methods for compound jamming recognition under different JNR conditions.

Figure 9 illustrates the trend curves of OA with respect to JNR for all methods on the test set from the compound jamming dataset. In general, all methods show a steady improvement in recognition performance as the JNR increases. However, the performance differences among methods are particularly pronounced in the low-JNR range, underscoring the greater difficulty of compound jamming recognition tasks. Notably, 1D-CNN and Transformer, both of which rely solely on time-domain information, perform poorly in compound jamming recognition. Their accuracy remains below 40% in the low JNR range of −10 dB to −4 dB, indicating near-complete failure under such conditions. This suggests that the additive nature of compound jamming components severely obscures discriminative features in the raw time-domain signal, making time-domain-only models ineffective at extracting meaningful information. It also reflects the inherent complexity of compound jamming in the feature space.

Additionally, 2D-CNN and DFCNN exhibit similar performance, with their accuracy curves plateauing around −4 dB and showing only marginal improvements thereafter. This may be attributed to the limitations in both 2D-CNN and DFCNN. As a relatively simple image-based model, 2D-CNN lacks the capacity to capture deeper structural features of compound jamming signals, resulting in an early performance bottleneck. DFCNN, although incorporating both time-domain and time–frequency branches, suffers from a similar issue. Its time–frequency branch, similar to that of 2D-CNN, struggles to extract deep features from time–frequency images. Furthermore, the absence of an effective fusion mechanism between the two domains constrains the overall representational capacity of the combined features, thereby preventing substantial performance gains. By comparison, DBRF-Net integrates DBB into its time–frequency branch, which significantly enhances the extraction of time–frequency features from compound jamming signals. It also utilizes AFFM to perform dynamic feature-level fusion, effectively leveraging the complementary strengths of multi-domain information. Experimental results demonstrate that DBRF-Net offers superior feature representation and robustness, enabling more accurate and reliable recognition in complex and dynamic compound jamming scenarios.

**Figure 10:**
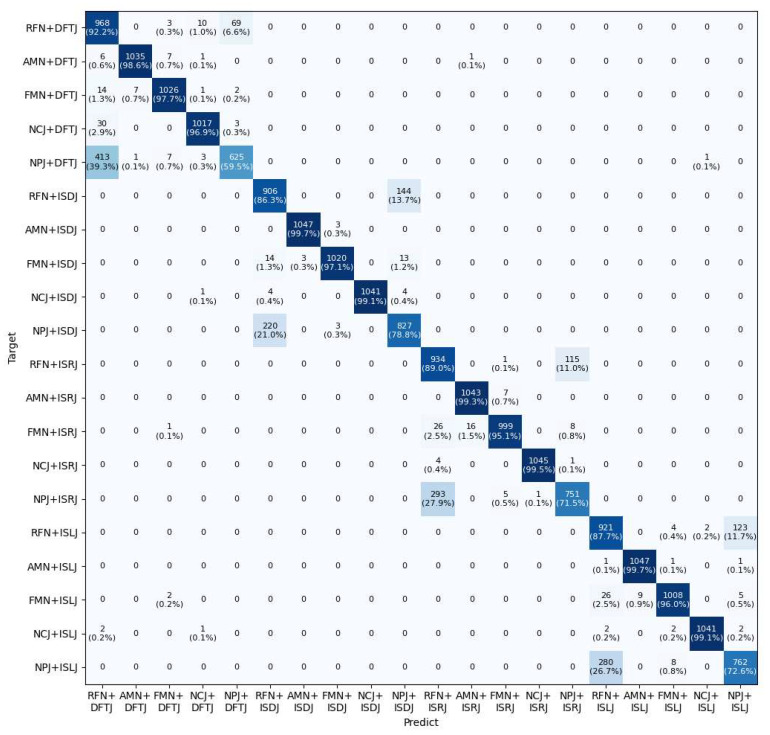
Confusion matrix of the proposed method on the compound jamming dataset.

Figure 10 shows the confusion matrix of the proposed method on the test set for the compound jamming recognition task. In Figure 10, the horizontal axis represents the predicted jamming categories, while the vertical axis denotes the ground-truth labels. Several notable misclassification patterns can be observed. In particular, significant confusion occurs between compound jamming classes such as NPJ + DFTJ and RFN + DFTJ, NPJ + ISDJ and RFN + ISDJ, NPJ + ISRJ and RFN + ISRJ, as well as NPJ + ISLJ and RFN + ISLJ. It is evident that these misclassifications are primarily associated with compound jamming signals involving NPJ and RFN. As shown in Figure 7, their time–frequency representations exhibit highly similar patterns, partly explaining the model’s difficulty in distinguishing them. This reflects a fundamental challenge in compound jamming recognition. Different jamming types may share overlapping features, particularly in the time–frequency domain. This feature similarity can significantly degrade classification accuracy.

Table 5 presents the comparison results of different methods on the test set from the compound jamming dataset in terms of OA, Macro-F1, and Kappa. Overall, DBRF-Net demonstrates consistently superior performance across all evaluation metrics, achieving an OA of 90.8%, a Macro-F1 of 0.908, and a Kappa of 0.903. These results highlight its comprehensive advantage in recognition accuracy, class-wise balance, and prediction consistency compared to existing methods. Among the baseline methods, JRNet performs relatively well, achieving 83.4%, 0.830, and 0.825 in OA, Macro-F1, and Kappa, respectively. Its competitive performance may be attributed to the inclusion of asymmetric convolution modules, which enhance the network’s ability to robustly capture subtle variations in compound jamming signals. Due to the increased structural complexity of compound jamming signals, their features are no longer as clearly separable as those in single jamming, making it more challenging to extract key discriminative features. Architectures with stronger generalization capacity and broader receptive fields are thus better suited to capturing such subtle differences. The proposed DBRF-Net addresses this challenge by not only integrating multi-modal information from both the time and time–frequency domains, but also by incorporating DBB and AFFM. These components enable the model to more effectively extract salient features from overlapping and structurally complex signals, contributing to its consistently superior performance across all three evaluation metrics.

In summary, DBRF-Net demonstrates leading performance across the entire JNR range for compound jamming recognition, with particularly strong recognition capability under low-JNR conditions. These experimental results fully validate the robustness and generalizability of the proposed method.

### 4.5. Ablation Study

We conducted ablation experiments to evaluate the contribution of different components of our model. First, we compared the performance of DBB and AFFM to evaluate their individual contributions. Then, we compared different fusion strategies to identify the most effective approach.

Effect of Removing Core Modules. To evaluate the individual contributions of the two key modules, DBB and AFFM, to the overall performance of DBRF-Net, three ablation models were constructed. All models were evaluated on the more challenging compound jamming dataset using the same training configuration. The first model removes the DBB (Without DBB), and the second excludes the AFFM (Without AFFM). The third model, by contrast, omits both DBB and AFFM (Without DBB & AFFM). Specifically, in the model without DBB, the original DBB module is replaced with a standard single-branch convolutional structure. This design is intended to evaluate the actual effectiveness of multi-scale feature extraction. In the model without AFFM, the features extracted from the time-domain and time–frequency branches are directly concatenated without adaptive fusion. This setting aims to assess the impact of the adaptive fusion mechanism. Finally, the performance of all three ablation models is compared with the original full model (Full Model).

Table 6 presents the OA comparison results of four models in the ablation study under different JNR conditions for compound jamming recognition. As shown in Table 6, the Full Model achieves the highest recognition accuracy across the entire JNR range from −10 dB to 10 dB. Its accuracy consistently outperforms the model without both key modules by 3% to 7%, demonstrating stronger overall robustness and recognition capability. Notably, at −10 dB, the Full Model reaches an accuracy of 70.5%, while the model Without DBB & AFFM achieves only 63.7%. This significant improvement indicates that DBB and AFFM play a critical role in enhancing noise-resistant recognition.

Further analysis shows that the model Without AFFM suffers a significant performance drop in the low and mid JNR range. This indicates that AFFM plays a key role in adaptively fusing time-domain and time–frequency features. The absence of AFFM leads to insufficient coordination between multi-domain features, which negatively affects the model’s discriminative performance. Similarly, the model Without DBB also performs poorly in the same JNR range, confirming the importance of DBB in multi-scale feature extraction and in enhancing the perception of weak patterns in compound jamming signals. In summary, the joint effect of DBB and AFFM is critical to improving the performance of the model.

**Figure 11:**
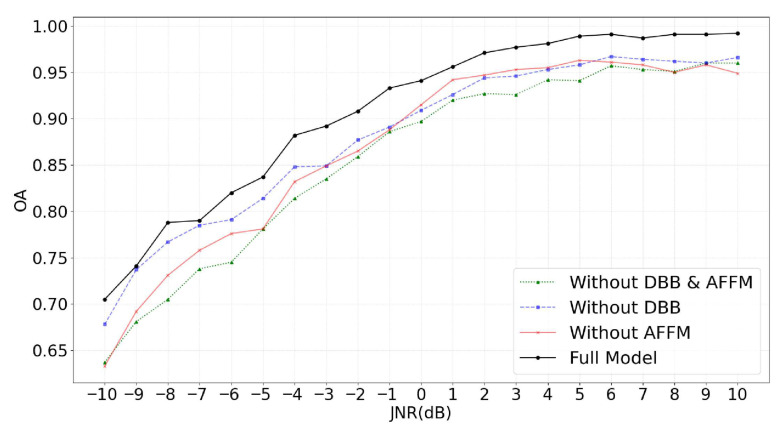
OA curves of the ablation study under different JNR conditions.

Figure 11 shows the trend curves of OA versus JNR for the four models in the ablation study on compound jamming recognition. Overall, the Full Model consistently achieves the highest accuracy across the entire JNR range. In contrast, the model Without DBB & AFFM performs the worst. The two models with only one module removed fall between these two extremes. This distribution further confirms the synergistic effect of DBB and AFFM within the network. Their collaboration effectively enhances the model’s ability to represent and recognize complex compound jamming signals, serving as a key foundation for the superior performance of DBRF-Net.

Fusion Strategy Comparison. Besides the ablation on individual modules, we further investigated the impact of different feature fusion mechanisms. Specifically, we compared (1) Without AFFM (direct concatenation), (2) Self-Attention Fusion, (3) cross-attention fusion, (4) GRU-based gating fusion, and (5) our proposed AFFM. In the Without AFFM strategy, the features extracted from the time-domain and time–frequency branches are directly concatenated without any explicit fusion module. In the Self-Attention Fusion strategy, the time-domain and time–frequency features are stacked as a two-token sequence and refined through a Transformer-style self-attention block, where each token attends to both itself and the other. The updated tokens are then combined by an attention pooling layer that assigns adaptive weights to yield a single fused representation. In the Cross-Attention Fusion strategy, the time-domain and time–frequency features exchange information through bi-directional cross-attention, and their updated representations are subsequently integrated using a channel-wise gating mechanism. In the GRU-based Gating Fusion strategy, the time-domain and time–frequency features are treated as a short sequence and processed by a gated recurrent unit (GRU). The final hidden state is taken as the fused representation, allowing the GRU to adaptively regulate the information flow between the two features. All fusion strategies were evaluated on the more challenging compound jamming dataset under the same training configuration.

Table 7 presents the OA comparison results of different fusion strategies for compound jamming recognition under various JNR conditions. As shown in the table, all the explicit fusion strategies yield clear improvements compared with the Without AFFM baseline. The proposed AFFM consistently delivers the best performance across the majority of JNR levels, while its performance in the remaining cases is nearly comparable to the best. Among the other fusion strategies, GRU-based Gating Fusion achieves the best performance after AFFM, followed by Cross-Attention Fusion and then Self-Attention Fusion. These results suggest that advanced, data-dependent fusion mechanisms such as attention and gating can indeed improve upon naive concatenation by enabling interaction or adaptive control between time-domain and time–frequency features. However, their effectiveness remains limited in this two-feature fusion setting. Self-Attention and Cross-Attention are originally designed for modeling long sequences, and applying them to the fusion of only two tokens (time-domain and time–frequency features) may introduce unnecessary complexity while failing to fully exploit their potential. The GRU-based Gating Fusion strategy is built upon GRU, which was originally designed to capture dependencies in long sequences. In our case of a very short sequence (two tokens), it functions as a gated fusion mechanism, providing adaptive control over the integration of the two features. However, its sequential update mechanism may favor the feature processed first, thereby introducing bias and reducing flexibility in balancing the two features.

In contrast, AFFM offers a lightweight but highly adaptive solution by directly learning a feature-wise weighting vector. This vector balances the relative contributions of time-domain and time–frequency representations in a data-driven manner. While attention or gating-based modules either underutilize their capacity in the two-token setting or introduce directional bias, AFFM achieves effective integration with only minimal additional complexity. It also delivers the most consistent gains across all JNR levels, particularly under low-JNR conditions. This indicates that the feature-wise weighting is more effective at preserving discriminative information when the signal is heavily corrupted by noise. Overall, these results demonstrate that AFFM strikes an effective balance between accuracy, robustness, and efficiency, making it a superior choice for multi-domain feature fusion in compound jamming recognition.

### 4.6. Computational Complexity and Runtime Evaluation

To evaluate the computational complexity and runtime performance of the proposed DBRF-Net, we employ five common metrics: Training time (s/epoch), Inference time (ms/sample), FLOPs (G), Parameters (M), and OA. We compare all previously mentioned models and introduce two additional variants of the proposed model: Without DBB and DBRF-Net(train). Specifically, in the Without DBB model, the original DBB module is replaced with a standard single-branch convolutional structure. This setup aligns with the Without DBB configuration in Section 4.5 (Ablation Study). In the DBRF-Net(train) model, the DBB module is used. However, no reparameterization is performed after training, and the multi-branch convolutional structure is retained during the inference phase. On the other hand, DBRF-Net(deploy) is the model proposed in this paper. It uses the DBB module and undergoes reparameterization into a single-branch convolutional structure after training. This re-parameterized model is then used for inference. All models were evaluated on the challenging compound jamming dataset using the same training configuration.

Table 8 presents the computational complexity and runtime performance of different methods for compound jamming recognition. From the table, DBRF-Net(deploy) does not show clear advantage in parameters, FLOPs, or training time compared to six representative baseline methods. Although it requires more parameters and longer training time, the deploy version achieves competitive inference efficiency and substantially higher recognition accuracy. Therefore, we argue that this additional computational cost is justified and essential for achieving its significantly improved OA. This improvement mainly comes from the incorporation of the DBB module and the reparameterization techniques, which enable DBRF-Net (deploy) to effectively capture subtle feature information in compound jamming signals. As a result, with parameter counts and computational overhead comparable to ResNet34 (21.44M vs. 21.4M, 7.98G FLOPs vs. 7.36G), DBRF-Net (deploy) achieves an OA of 90.8%, which is 15.6 percentage points higher than ResNet34.

Additionally, by comparing the last three columns of Table 8, it can be observed that the re-parameterized DBRF-Net(deploy) maintains the same OA as DBRF-Net(train) (90.8%) while significantly reducing parameters, FLOPs, and inference time. At the same time, these computational costs of DBRF-Net(deploy) are comparable to those of the standard convolutional model Without DBB, while its OA is 2.7% higher. This strongly demonstrates that after training, the multi-branch convolutional structure can be equivalently re-parameterized into a single-branch convolution for deployment, allowing the model to retain its high recognition accuracy while reducing parameters, FLOPs, and inference time. Although the multi-branch convolution introduces additional computational overhead during training for improved performance, the model can be re-parameterized into a standard convolution for deployment, achieving a lossless improvement in recognition accuracy during inference.

## 5. Conclusions

In this article, we proposed DBRF-Net, a dual-branch residual fusion network for the recognition of radar active jamming signals. The model is designed to classify nine typical single jamming types and twenty compound jamming combinations under various JNR conditions. It simultaneously processes both time-domain sequences and time–frequency representations, thereby enriching the feature space and enhancing the expressive capacity of jamming features. In addition, the incorporation of DBB and AFFM further strengthens the network’s ability to extract multi-scale features and perform adaptive cross-domain fusion. Extensive experiments demonstrate that DBRF-Net achieves superior performance across a wide range of JNR conditions. In the single jamming recognition task, it outperforms baseline methods throughout the −20 dB to 5 dB range, reaching 70.7% accuracy at −16 dB, 91.1% at −12 dB, and 98.9% at −6 dB. In the more complex compound jamming scenario, DBRF-Net maintains excellent performance even under low JNR conditions, with accuracy reaching 70.5% at −10 dB, 90.8% at −2 dB, and 99.0% at 6 dB. Ablation experiments further validate the effectiveness of the DBB and AFFM. The removal of either module results in significant performance degradation, especially under low JNR conditions. This confirms the complementary roles of DBB and AFFM in jointly enhancing the robustness of recognition.

Overall, DBRF-Net demonstrates accurate and stable recognition capabilities across both simple and complex jamming scenarios, especially under low JNR conditions. This work provides a promising foundation for advancing intelligent radar anti-jamming technologies. Future research will focus on broader jamming types, more challenging signal environments, and more efficient real-time deployment architectures to meet the growing demands of modern electromagnetic confrontation.

## Figures and Tables

**Table 1:** Simulation parameters of jamming signals.

Jamming Type	Parameters	Range of Values
RFN	Carrier frequency	15–60 MHz
	Bandwidth	15–40 MHz
AMN	Carrier frequency	15–60 MHz
FMN	Bandwidth	15–40 MHz
NCJ	Carrier amplitude modulation index	0.4–1
NPJ	Signal duration	20 μs
DFTJ	Carrier frequency	15–60 MHz
	Bandwidth	20–80 MHz
	False target number	3–6
	False target delay	1.5–5 μs
ISDJ ISRJ ISLJ	Carrier frequency	15–60 MHz
	Bandwidth	15–40 MHz
	Signal duration	20 μs
	Slice width	1 μs
Compound jamming	Same as constituent single jamming components	\

**Table 2:** OA (%) of different methods for single jamming recognition under various JNR levels.

Model	−20	−18	−16	−14	−12	−10	−8	−6	−4	−2	0	2	4	Average
1D-CNN	35.1	41.3	39.1	38.9	46.7	58.4	69.1	76.9	83.8	89.1	90.2	92.9	93.8	67.0
2D-CNN [32]	21.3	30.9	45.8	60.2	69.8	76.9	81.6	87.6	91.6	93.1	93.1	96.2	96.4	74.6
Transformer [33]	18.0	20.7	31.3	31.8	37.8	48.7	55.3	68.9	76.2	82.0	86.9	88.9	90.7	58.3
ResNet34 [25]	25.8	34.2	56.9	69.1	79.6	83.8	87.1	88.7	90.9	94.9	96.0	96.9	98.0	79.1
JRNet [20]	18.2	24.9	43.3	57.1	64.4	75.6	83.6	88.4	93.6	97.1	96.9	95.6	95.3	74.1
DFCNN [22]	47.8	57.3	66.2	74.0	81.6	85.1	88.9	90.7	92.7	95.3	95.1	96.9	96.4	83.6
DBRF-Net (ours)	**53.1**	**67.8**	**70.7**	**81.1**	**91.1**	**93.1**	**97.1**	**98.9**	**99.3**	**99.8**	**99.8**	**99.8**	**99.8**	**89.7**

**Table 3:** OA, Macro-F1, and Kappa of different methods for single jamming recognition.

	1D-CNN	2D-CNN	Transformer	ResNet34	JRNet	DFCNN	DBRF-Net (Ours)
OA (%)	67.0	74.6	58.3	79.1	74.1	83.6	**89.7**
Macro-F1	0.665	0.748	0.576	0.794	0.749	0.836	**0.897**
Kappa	0.629	0.715	0.534	0.765	0.710	0.816	**0.884**

**Table 4:** OA (%) of different methods for compound jamming recognition under various JNR levels.

Model	−10	−8	−6	−4	−2	0	2	4	6	8	10	Average
1D-CNN	11.4	17.1	23.4	35.0	46.5	57.4	67.8	72.4	76.1	77.5	79.4	51.7
2D-CNN	42.0	55.2	61.4	64.9	66.7	67.6	68.2	68.3	67.3	68.6	68.4	64.0
Transformer	11.9	15.5	21.1	31.4	38.1	48.0	51.2	55.1	59.1	60.1	61.4	40.9
ResNet34	52.4	66.3	73.6	75.2	77.4	79.1	78.8	78.5	79.7	79.2	79.4	75.2
JRNet	55.4	69.4	77.5	81.2	84.7	87.1	90.4	91.0	92.0	91.8	91.9	83.4
DFCNN	47.6	59.3	63.8	67.1	68.1	68.4	69.3	68.3	69.8	69.6	69.7	66.0
DBRF-Net (ours)	**70.5**	**78.8**	**82.0**	**88.2**	**90.8**	**94.1**	**97.1**	**98.1**	**99.1**	**99.1**	**99.2**	**90.8**

**Table 5:** OA, Macro-F1, and Kappa of different methods for compound jamming recognition.

	1D-CNN	2D-CNN	Transformer	ResNet34	JRNet	DFCNN	DBRF-Net (Ours)
OA (%)	51.7	64.0	40.9	75.2	83.4	66.0	**90.8**
Macro-F1	0.517	0.636	0.412	0.737	0.830	0.655	**0.908**
Kappa	0.492	0.621	0.375	0.739	0.825	0.642	**0.903**

**Table 6:** OA (%) of the ablation study under various JNR levels.

Model	−10	−8	−6	−4	−2	0	2	4	6	8	10	Average
Without DBB&AFFM	63.7	70.5	74.5	81.4	85.9	89.7	92.7	94.2	95.7	95.1	96.0	85.8
Without DBB	67.8	76.7	79.1	84.8	87.7	90.9	94.4	95.3	96.7	96.2	96.6	88.1
Without AFFM	63.3	73.1	77.6	83.2	86.5	91.5	94.7	95.5	96.1	95.0	94.9	87.0
Full model	**70.5**	**78.8**	**82.0**	**88.2**	**90.8**	**94.1**	**97.1**	**98.1**	**99.1**	**99.1**	**99.2**	**90.8**

**Table 7:** OA (%) of different fusion strategies for compound jamming recognition under various JNR levels.

Fusion Strategy	−10	−8	−6	−4	−2	0	2	4	6	8	10	Average
Without AFFM	63.3	73.1	77.6	83.2	86.5	91.5	94.7	95.5	96.1	95.0	94.9	87.0
Self-Attention Fusion	66.9	75	79.3	84.3	88.2	93.5	94.4	96.2	96.7	95.3	95	88
Cross Attention Fusion	65	76.6	79.6	84.5	**90.9**	92.2	95.8	96.5	97	97	97.4	88.7
GRU-based Gating Fusion	65.6	74.7	78.6	83.8	90.1	93.6	**97.2**	97.5	98.1	97.6	97.7	89
AFFM (ours)	**70.5**	**78.8**	**82.0**	**88.2**	90.8	**94.1**	97.1	**98.1**	**99.1**	**99.1**	**99.2**	**90.8**

**Table 8:** Computational complexity and runtime performance of different methods for compound jamming recognition.

	1D-CNN	2D-CNN	Transformer	ResNet34	JRNet	DFCNN	Without DBB	DBRF-Net (Train)	DBRF-Net (Deploy)
Training time (s/epoch)	7.5	94.5	21.6	106.1	104.7	100.6	111.9	149.0	149.0
Inference Time (ms/sample)	0.24	0.28	0.18	3.66	1.57	0.53	1.47	12.75	1.24
FLOPs (G)	0.32	2.71	0.28	7.36	3.63	3.36	8.01	16.87	7.98
Parameters (M)	0.16	0.57	0.37	21.4	11.18	1.15	21.45	47.30	21.44
OA (%)	51.7	64.0	40.9	75.2	83.4	66.0	88.1	90.8	90.8

## Data Availability

The raw data supporting the conclusions of this article will be made available by the authors on request.
